# Contamination Assessment and Potential Human Health Risks of Heavy Metals in Urban Soils from Grand Forks, North Dakota, USA

**DOI:** 10.3390/toxics11020132

**Published:** 2023-01-29

**Authors:** Muhammad Saleem, Donald A. Sens, Seema Somji, David Pierce, Yuqiang Wang, August Leopold, Mohammad Ehsanul Haque, Scott H. Garrett

**Affiliations:** 1 Department of Pathology, School of Medicine and Health Sciences, University of North Dakota, Grand Forks, ND 58202, USA; 2 Department of Chemistry, University of North Dakota, Grand Forks, ND 58202, USA

**Keywords:** urban soil, heavy metals, risk assessment, ecological risk, pollution, carcinogenic risk

## Abstract

Heavy metal (HM) pollution of soil is an increasingly serious problem worldwide. The current study assessed the metal levels and ecological and human health risk associated with HMs in Grand Forks urban soils. A total 40 composite surface soil samples were investigated for Mn, Fe, Co, Ni, Cu, Zn, As, Pb, Hg, Cr, Cd and Tl using microwave-assisted HNO_3_-HCl acid digestion and inductively coupled plasma mass spectrometry (ICP-MS) analysis. The enrichment factor (EF), contamination factor (CF), geoaccumulation index (Igeo), ecological risk and potential ecological risk index were used for ecological risk assessment. The park soils revealed the following decreasing trend for metal levels: Fe > Mn > Zn > Cr > Ni > Cu > Pb > As > Co > Cd > Tl > Hg. Based on mean levels, all the studied HMs except As and Cr were lower than guideline limits set by international agencies. Principal component analysis (PCA) indicated that Mn, Fe, Co, Ni, Cu, Zn, As, Cd, Pb, Cr and Tl may originate from natural sources, while Hg, Pb, As and Cd may come from anthropogenic/mixed sources. The Igeo results showed that the soil was moderately polluted by As and Cd and, based on EF results, As and Cd exhibited significant enrichment. The contamination factor analysis revealed that Zn and Pb showed moderate contamination, Hg exhibited low to moderate contamination and As and Cd showed high contamination in the soil. Comparatively higher risk was noted for children over adults and, overall, As was the major contributor (>50%), followed by Cr (>13%), in the non-carcinogenic risk assessment. Carcinogenic risk assessment revealed that As and Cr pose significant risks to the populations associated with this urban soil. Lastly, this study showed that the soil was moderately contaminated by As, Cd, Pb and Hg and should be regularly monitored for metal contamination.

## 1. Introduction

With rapid urbanization and industrialization, urban soil pollution is becoming a serious problem worldwide and continues to attract increasing attention [1,2,3,4,5,6,7,8]. Soil management is a major component of agricultural food production, and soil serves as a sink for toxic substances, such as HMs [9,10,11]. Urban soils, including residential and park soils, which are the most common types of urban soils, expose local populations to HMs, and their contamination may have a direct effect on human health. Thus, the quality of urban soils is of great concern for human health [11,12,13,14,15]. It is vital for local states and local governments, as well as other stakeholders, to identify possible sources of contamination, assess the risk of HM soil pollution and propose solutions to remediate the soils in the investigated areas.

Heavy metals are some of the most common pollutants in the environment and have received much attention due to their toxicity, persistence and complex geochemical properties [15,16]. Heavy metals are released into the environment from natural and anthropogenic sources (burning of fossil fuels, agricultural activities, vehicle emissions, paint erosion, atmospheric deposition, industrial activities and soil weathering) [4,8,17,18,19,20]. Humans are exposed to HMs in urban soils via hand-to-mouth ingestion, dermal contact and inhalation [21,22], and they pose a risk to human health. Therefore, assessment of HMs and their sources in urban soils is important for pollution control and urban planning.

Heavy metals are distinctive elements of the Earth’s crust. However, anthropogenic activities can change their natural balance and chemical cycles in the environment. Since they cannot be biologically degraded, they persist in nature for an indefinite time and, as a result, gradually accumulate to high levels in soil [23,24,25]. They can enter the human body fortuitously via any route and pose a threat to human health [18]. The effects of heavy metals on human health are considerable. For example, cadmium exposure can lead to kidney disease, lung cancer, osteoporosis and other chronic diseases [25,26,27]. Nickel can cause lung and prostate cancers [28]. Lead is reported to be a human carcinogen. In addition, exposure to it may affect the nervous system, especially in children, causing permanent learning disabilities and behavior disorders. It can cause miscarriages in pregnant women, severely damage the brain and kidneys of developing fetuses and, ultimately, causing death [26,27,28]. Mercury is regarded as more toxic than other non-essential metals [29,30] and can cause cancer [31,32] and a variety of other disorders affecting the immune system, heart, kidneys, lung and liver [29,33,34,35,36,37,38]. Essential elements at high levels can also have toxic effects [39]. For example, zinc is an essential element but can affect the nervous system, disrupt homeostasis and affect an individual’s IQ at high levels [25]. Similarly, manganese, an essential nutrient, can cause neurological disorders and affect the lungs at high levels [40]. In order to assess the environmental impact of heavy metals on human health, it is necessary to comprehensively assess their levels in the environment. Therefore, this study aimed to: (a) assess As, Cd, Co, Cr, Cu, Fe, Mn, Pb, Ni, Tl and Zn levels in urban soils; (b) compare metal levels with international soil guidelines and worldwide reported levels; (c) assess possible sources using principal component analysis; (d) assess the ecological risk; and (e) investigate the potential risk of heavy metal soil contamination for human health.

## 2. Materials and Methods

### 2.1. Study Area

Grand Forks (47.0329° N, 97.0329° E) is located in northeast North Dakota, USA, and is one of the oldest cities and the third most highly populated in the state. It lies within the Red River Valley in North Dakota and is part of the Red River basin. The valley is blanked by lacustrine deposits that developed from the glacial Lake Agassiz. Grand Forks has a sub-humid continental climate and significant variation in temperatures from summer to winter. Blizzard conditions with snow and strong winds occur several times each winter [41]. According to the 2020 census, the city’s population is almost 60,000. The city is an important center of transportation, health care and education and is the location of industrial activities, such as agribusiness, manufacturing, etc. The soils of Grand Forks county primarily comprise poorly drained clay and silt, with relatively low permeability levels. Farming is the major livelihood in this area, and corn, soybeans and spring wheat are the most common crops [42,43]. 

### 2.2. Sample Collection and Processing

From August to September 2021, a total 40 composite soil samples (park samples: 30; residential samples: 10; 1–20 cm depth) were collected by systematic random sampling from the parks and residential areas in Grand Forks, North Dakota, USA [44]. Four to eight sub-samples were collected around the same location and mixed thoroughly to obtain a composite sample. Samples were collected in pre-cleaned zip-lock bags and transferred to the laboratory. Stones, gravel, plant residues and coarse particles were removed manually. Samples were dried, ground, homogenized with an agate pestle and mortar and stored for metal analysis [45,46,47]. 

### 2.3. Physicochemical Characteristics and Elemental Analysis

For pH, electrical conductivity (EC) and total dissolved solids (TDS), soil and water suspensions were used [48,49]. For metal analysis, 0.5 g of dried soil samples was digested in a microwave with 4.5 mL HNO_3_ and 1.5 mL HCl [50]. The digested sample was centrifuged and filtered and the volume was adjusted to 50 mL with deionized water. Further dilution was carried out before analysis if deemed necessary. In the present study, the samples were analyzed for As, Cd, Co, Cr, Cu, Fe, Mn, Pb, Ni, Tl and Zn using ICP-MS (Thermo Scientific iCAP Qc) in kinetic energy discrimination mode (KED). All operating parameters were optimized to meet requirements as defined by the manufacturer prior to method calibration and analysis. Mercury was analyzed with a Milestone DMA-80 Tri Cell direct mercury analyzer (Shelton, CT) equipped with a 40 position auto-sampler by following the instructions given by the manufacturer. All measurements were undertaken in triplicate. Quantification of the metals in various samples was undertaken with the calibration line method, maintaining the optimum analytical conditions. Quality assurance and quality control were assessed using duplicate samples, reagent blanks, method blanks, spikes and a standard reference material (NIST 2711a). All the reagents/standards were prepared in deionized water. All the glassware was soaked in 10–20% (*v*/*v*) HNO_3_ for at least 24 h, rinsed with distilled water and oven-dried before use [51]. 

### 2.4. Estimation of Pollution Index

Soil quality can be estimated from numerous factors/indices, as reported in the literature [52,53]. The pollution index of soil can be quantified in terms of the enrichment factor (EF), contamination factor (Cf), geoaccumulation index (Igeo), ecological risk (Ei) and potential ecological risk index (RI), and all these were calculated as detailed below. 

#### 2.4.1. Enrichment Factor (EF)

The concept of an EF was established to differentiate between the natural and anthropogenic contributions of metals in soil, and it is the most important tool for the assessment and differentiation of the contamination levels resulting from heavy metal pollution. It was calculated using the following relationship [54,55,56,57]:

(1) $$EF=\frac{{\left[X/M_{ref}\right]}_{smaple}}{{\left[X/M_{ref}\right]}_{crust}}$$

where [X/Mref]sample and [X/Mref]crust refer to the ratio of the mean concentrations (μg/g) of the target metal in the examined soil and the continental crust, respectively. The EF is the standardization of the level of the target element in a soil sample by the reference element. For this study, the Fe level in the soil was selected as the reference element and the background Earth crust values as given by Lide (2005) were used [58]. EF values were interpreted in various categories, as suggested by Sutherland (2000) [59].

#### 2.4.2. Contamination Factor (Cf)

The potential contamination of soil can be assessed using the contamination factor. The Cf is the ratio between the metal concentrations in the examined soil and in pre-industrial soil and is computed as follows:

(2) Cf=CnCb

where Cn and Cb refer to the mean concentrations of a metal in soil and the Earth’s crust, respectively [51,60,61,62]. Cf values were interpreted in various categories, as suggested by Sur et al. (2022) [63].

#### 2.4.3. Geoaccumulation Index (Igeo)

The Igeo was initially proposed by Muller (1969) [64] and has become one of the most widely used quantitative indices in assessing pollution levels since it takes the effects of human activities into consideration [65,66,67]. It is computed as follows:

(3) $$I_{geo}={log}_{2}(\frac{C_{n}}{1.5B_{n}})$$

where Igeo is the index of geoaccumulation; C_n_ is the heavy metal concentration measured in soils; and B_n_ is the geochemical background value of the corresponding toxic element in the soil [58].

#### 2.4.4. Ecological Risk (Ei) and Potential Ecological Risk Index (RI)

The ecological risk and the potential ecological risk index were developed by Hakanson (1980) [60]. The ecological risk permits the assessment of the toxic risk of individual heavy metals and is computed as follows:

(4) $$E_{i}=T\times C_{f}$$

where T is the toxic factor of the heavy metal i (As = 10, Cd = 30, Cr = 2, Cu = 5, Mn = 1, Ni = 5, Pb = 5, Zn = 1) and Cf is the contamination factor of the heavy metal i [55,68,69]. The potential toxicity of the heavy metals causing the ecological risk can be assessed using the RI [69,70,71]. The Ei and RI values can be interpreted in various categories, as described by Sur et al. (2022) [63]. The RI is calculated as the sum of the Ei for each heavy metal using the following equation:

(5) $$RI=\sum_{i=1}^{n}E_{i}$$

### 2.5. Statistical Analysis

All results were subjected to both univariate and multivariate statistical analyses using Excel and SPSS Statistics. The relationships related to the heavy metal values of the samples were evaluated using principal component analysis (PCA) [72]. PCA is a common statistical analysis method that simplifies the measured analytical data into latent factors by integrating multiple indicators into multiple composite indicators with a minimal loss of information. It is widely utilized with correlation analysis to categorize possible metals sources [73,74]. Principal components with eigenvalues greater than 1 were retained and the Kaiser–Meyer–Olkin (KMO) method was employed to assess the suitability of the data for PCA [75].

### 2.6. Human Health Risk Assessment of Heavy Metals

The human health risk of the heavy metals was assessed using the method recommended by the U.S. Environmental Protection Agency. Non-carcinogenic risk (NCR) and carcinogenic risk (CR) were considered with this method. Human exposure to metals can occur through three main pathways: (i) oral ingestion via the mouth, (ii) inhalation via the nose and (iii) dermal absorption via the skin. Among these routes, only the ingestion route, which is usually significant, was considered in the current study in both the NCR and CR assessments. To assess the CR and NCR from ingesting soil in children and adults, the average daily exposure dose via ingestion (ADDing) of heavy metals was measured, following which risk characteristics were evaluated. The non-carcinogenic risk hazard quotient (HQ) and the carcinogenic risk were calculated via reference doses (RfDs) and the cancer slope factor (CSF), respectively. To determine the overall non-carcinogenic risk posed by all the metals, the hazard index (HI) was calculated (Equations (6)–(9)) [74,76]:

(6) $$ADD_{ing}=\frac{C\times IR\times CF\times EF\times ED}{BW\times AT}$$

(7) $$HQ_{ing}=\frac{ADD_{ing}}{RfD}$$

(8) $$HI=\sum_{i=1}^{n}HQ_{ing}$$

(9) $$CR=ADD_{\mathrm{ing}}\times CSF$$

where C is the mean concentration of the metals in soil (mg/kg); IR is the ingestion rate (100 mg/day for adults and 200 mg/day for children); CF is the conversion factor (10^−6^, kg/mg); EF is the exposure frequency (350 days/year); ED is the exposure duration (24 years for adults and 6 years for children); BW is the body weight (70 kg for adults and 15 kg for children); AT is the average time (365 × ED days for non-carcinogenic risk and 72  ×  365 days for carcinogenic risk); and CSF represents the cancer slope factor (mg/kg.day) [74,76]. 

## 3. Results and Discussion

### 3.1. Chemical Characteristics

The physicochemical characteristics of the soil (pH, electrical conductivity (EC) and total dissolved solids (TDS)) were determined from the water extract. Soil pH is an important soil parameter for the assessment of the availability of nutrients, and its variation affects the availability of nutrients. The results shown in Table 1 indicate that the pH of park soil samples ranged from 6.35 to 7.03, with a mean of 6.75. Similarly, the EC and TDS showed ranges from 128.3 to 1417 µS/cm and 85.5 to 945.0 mg/L, with average values of 336.1 µS/cm and 223.1 mg/L, respectively. Similarly, the mean pH, EC and TDS in residential soil were 6.70, 281.9 and 187.9, respectively. Overall, based on the average pH, the soil samples were near neutrality. A near-neutral pH can improve the cation exchange capacity (CEC) and base cation bio-availability, which is responsible for soil fertility [77,78]. Similarly, the soluble salt concentrations were found to be normal and within the safe range (EC < 1000 µS/cm) [79]. 

### 3.2. Metal Levels and Comparison with Worldwide Reported Levels and Guidelines

Descriptive statistics for the HM levels in park soils and residential soils in Grand Forks are shown in Table 1. For the studied metals in park soils, Fe showed the highest mean concentration (21,011 μg/g), followed by Mn (818.3 μg/g) and Zn (79.99 μg/g), while Cd (0.665μg/g), Tl (0.365 μg/g) and Hg (0.062 μg/g) showed the lowest mean values. Overall, the following decreasing trend was observed in park soils: Fe > Mn > Zn > Cr > Ni > Cu > Pb > As > Co > Cd > Tl > Hg. The mean concentrations (Table 1) of Mn, Fe, Co, Ni, Cu, Zn, As, Pb, Hg, Cr, Cd and Tl in residential soil were 783.5 μg/g, 20,896 μg/g, 6.708 μg/g, 24.80 μg/g, 17.38 μg/g, 81.58 μg/g, 9.147 μg/g, 14.67 μg/g, 0.060 μg/g, 23.62 μg/g, 0.670 μg/g and 0.377 μg/g, showing a decreasing trend almost similar to that observed in park soils. To determine the average variation in the heavy metal levels, the coefficient of variation (CV) was calculated. In general, a CV > 35% indicates high variation, 15% < CV < 35% reflects moderate variation and CV ≤ 15% is generally considered low variation. According to our analysis, the CVs of most of the studied metals in park soils showed a low level of spatial variation (CV ≤ 15%) and a uniform spatial distribution, except Pb, Hg, Cr and Mn. Pb (56%) and Hg (55%) indicated higher variation, while Mn (17%) and Cr (17%) showed moderate-level variations. Similarly, in residential soil, Fe, Co, Zn, Cr and Cd revealed low levels of spatial variability; Mn, Ni, Cu, As and Tl showed moderate variations; and Pb (43%) and Hg (36%) exhibited greater variations. These results indicate that greater variability existed in the heavy metal distribution and spatial heavy metal distribution in residential areas, and they were less homogeneous than that in park soil [80,81]. Overall, the magnitude of the CVs in soil showed the following order: Zn < Fe < Co < Cd < Tl < Cr < Cu < Ni < As < Mn < Hg < Pb. This indicated that the heavy metal distribution may be lithogenic. Moreover, the higher CV levels for Pb and Hg also revealed that the soil was influenced by discrete sources associated with anthropogenic activities and natural or mixed sources [65]. 

Heavy metal levels in park soils and residential soils were compared with guidelines set by international agencies (California Human Health (SSLs), Dutch Soil Guidelines, Canadian Soil Quality Guidelines, Spanish Government recommendations, the Government of Armenia) (Table 2). Based on average values, in all studies, the HM levels were lower than the guideline limits set by the agencies, except those of As and Cr. Both the As and Cr mean levels were found to be higher than the levels set by California Human Health (SSLs) for residential areas. A comparison of HM levels in the soil of Grand Forks with the levels found in other cities worldwide (China, Pakistan, Italy, Iran, India, Armenia, Greece, Chile, Portugal, Nigeria, USA, Norway, Mongolia) is shown in Table 2. The comparison analysis (Table 2) indicated that Co, Cu, Hg, Tl, Zn, Ni, Pb and Fe levels were almost lower than the reported worldwide values. Concentrations of Cr in the examined soil were much lower than those reported in most of the other cities, except Karachi, Caserta, Hualpén and Niger. Cadmium levels were notably higher than the levels reported in Ghaziabad, Shanghai and Oslo. However, the As mean levels were higher than the reported levels in Yerevan, Shanghai, Hualpén, Lisbon, Oslo and Darkhan and lower than those from Jiaozuo city, Caserta, Isfahan city and Chicago. Similarly, Mn levels were also found to be higher than most of the other cities, except for Caserta and Thiva. The differences in the metal levels might have been due to different sampling strategies and measuring methods and other activities [82]. 

**Figure 1:**
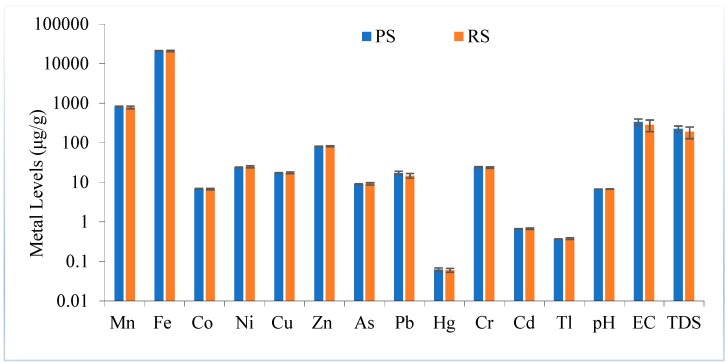
Comparison of average metal levels (μg/g, ±SE) in acid extracts of park soils (PSs) and residential soils (RSs).

A comparison of the average metal levels in park and residential soils is shown in Figure 1. The levels of metals were almost similar in both areas. Overall, the levels of Fe, Mn and Zn were high, whereas the levels of Cd, Tl and Hg were the lowest in park and residential soils.

### 3.3. Principal Component Analysis (PCA)

One of the most important parts of the current study was the source apportionment for the metal pollutants in the urban soil. For this purpose, PCA was employed in order to assess the metal content relationships and the metal source apportionment for the soils. The Kaiser–Meyer–Olkin (KMO) test exhibited a good value (0.817), indicating that the HM data from urban soils were suitable for PCA. 

**Figure 2:**
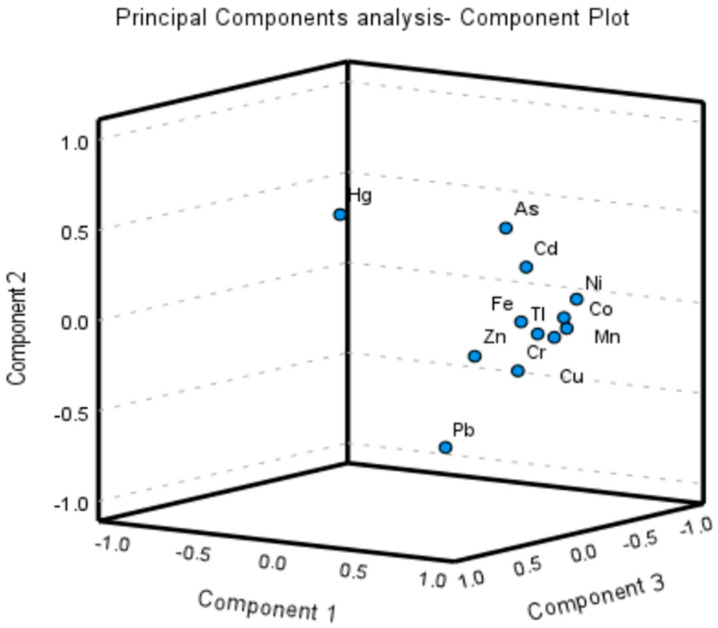
Component plot for heavy metal levels in the soil obtained from principal component analysis.

The PC loadings of the heavy metals in the soil samples are shown in Table 3 and in the component plot diagram (Figure 2). Three PCs were extracted with eigenvalues > 1, together explaining more than 77% of the total variance in the data, as shown in Table 3. The first principal component (PC 1) revealed that 58.65% of the total variance was explained by the highest loadings for Mn, Fe, Co, Ni, Cu, Zn, As, Cd, Pb, Cr and Tl; PC 2 showed significant loadings for As, Hg and Pb (inverse loading); and PC 3 indicated a higher loading for Hg. PC1 explained 58.65% of the total variance, and the metal concentrations mostly exhibited the smallest CV values (i.e., < 15%), which indicated that these metals might originate from natural sources, such as lithogenic components and soil parent materials [45,102,103]. Similarly, PC2 and PC3 indicated anthropogenic sources. Hg and Pb manifested the highest coefficients of variation compared to the other studied metals (see also the component plot), indicating that human activities were the main sources of these metals in the soils. Overall, Hg, Pb, As and Cd intrusion in the soil might have been due to various human activities, such as coal combustion, vehicle exhausts, automobile brakes, vehicle tires, pesticides and chemical fertilizers [104,105,106,107,108,109,110,111,112]. 

### 3.4. Ecological Risk Assessment

**Figure 3:**
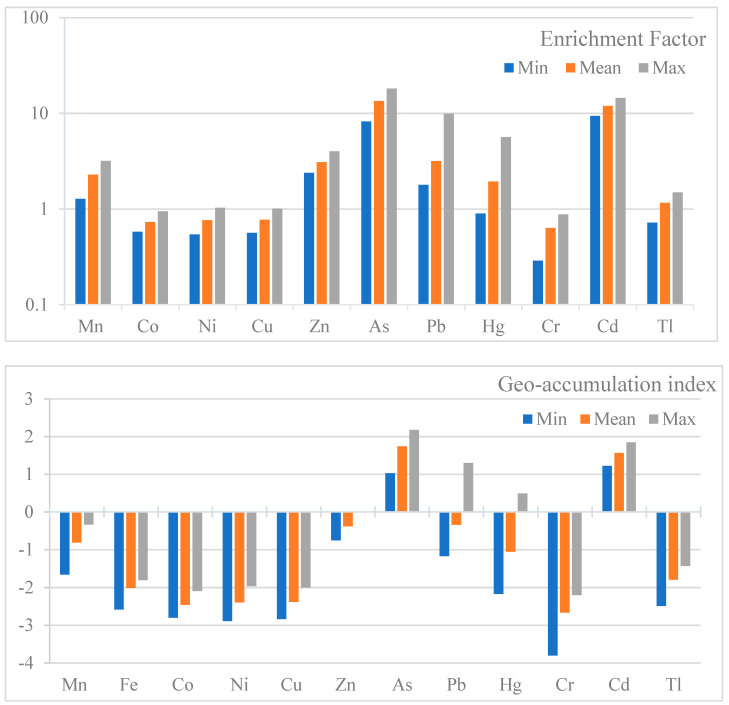

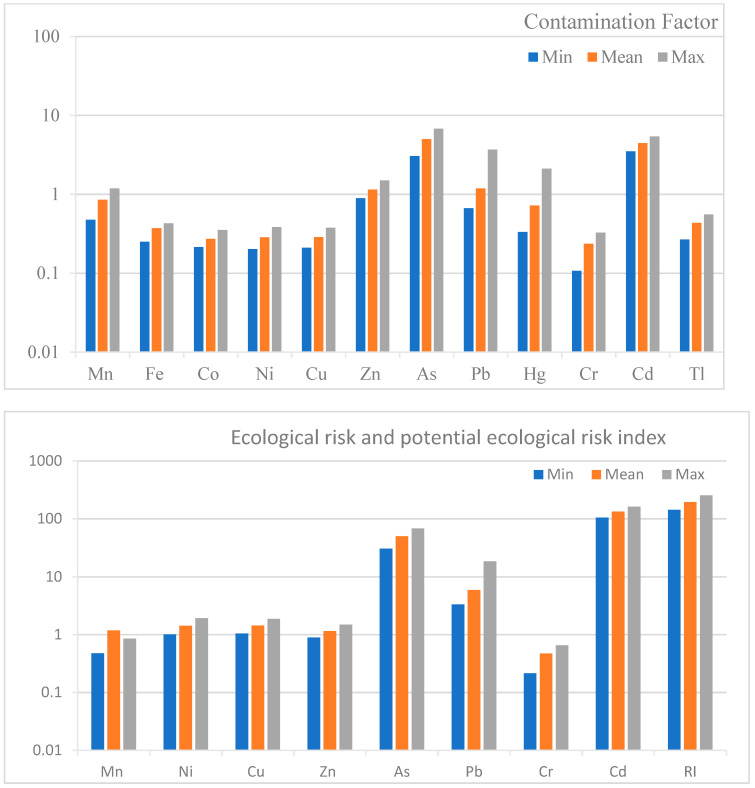
Risk assessment for urban soils using the enrichment factor, geoaccumulation factor, contamination factor, ecological risk and potential ecological risk index.

To determine the ecological risk of the urban soil, risk assessments were undertaken using the enrichment factor, contamination factor, geoaccumulation index and potential ecological risk assessment. The enrichment factor values for Mn, Co, Ni, Cu, Zn, As, Pb, Hg, Cr, Cd and Tl were in the ranges (mean values in bracket) of 1.28–3.19 (2.29), 0.58–0.94 (0.73), 0.54–1.03 (0.76), 0.56–1.01 (0.77), 2.39–4.01 (3.08), 8.22–18.18 (13.43), 1.79–3.17 (9.90), 0.90–5.66 (1.94), 0.29–0.88 (0.63) and 0.72–1.49 (1.16), respectively. Based on mean levels, Co, Ni, Cr, Tl and Cu showed minimum enrichment; Mn, Zn, Pb and Hg showed moderate enrichment; and As and Cd exhibited significant enrichment. Similarly, Pd and Hg also exhibited significant enrichment from the maximum concentrations measured in the samples. The contamination factors were also calculated to evaluate the soil contamination, and their concentration variations are shown in Figure 3. The mean Cf values for Co, Ni, Cu, Cr, Tl and Fe showed low contamination (Cf < 1); Zn and Pb showed moderate contamination; Hg exhibited low–moderate contamination; and As and Cd showed considerable contamination in soil. Overall, the following decreasing order was calculated for the Cf on the basis of mean levels: Cr < Co < Ni < Cu < Fe < Tl < Hg < Mn < Zn < Pb < Cd< As. The Igeo values for the heavy metals in the soil are shown in Figure 3. The mean Igeo decreased in the soil in the following order: Cr < Co < Ni < Cu < Fe < Tl < Hg < Mn < Zn < Pb < Cd< As. Manganese, Fe, Co, Ni, Cu, Zn, Cr and Tl were in the unpolluted category; As and Cd showed moderate pollution; and Pb and Hg ranged from unpolluted to moderately polluted. The ecological risk index for the metals was also evaluated (Figure 3). The results showed that the elements had low-contamination status, except for As and Cd. Moderate risk was associated with As, while Cd manifested high risk. Overall, the potential risk from heavy metals in the soils was measured with the potential ecological risk index (RI), which revealed moderate risk for the metal levels in the soils on a cumulative basis

### 3.5. Health Risk Assessments

The non-carcinogenic and carcinogenic risk assessments for children and adults were determined via the oral pathway (Table 4). The hazard quotients (HQs) for the HMs for adults and children exhibited the following trend: Hg < Zn < Co < Cu < Cd < Ni < Pb < Tl < Mn < Cr < As. Comparatively higher risk was noted for children over adults, which was due to higher soil ingestion rates and smaller body weights [113,114,115,116,117]. The HQs for As, Cr, Mn, Tl and Pb were 54%, 14%, 10%, 8% and 7%, respectively, for adults and children. On the other hand, the HQs for the other HMs accounted for < 7% of the hazard index (HI) values. The HI values were 7.63 × 10^−2^ and 7.12 × 10^−1^ for adults and children, respectively, which were less than 1, indicating that there was no non-carcinogenic risk on a cumulative basis. Overall, As was the major contributor (>50%) for non-carcinogenic risk, followed by Cr (>13%). The carcinogenic risk (CR) from As, Cd, Cr and Pb was also assessed (Table 4). A CR value < 10^−6^ was considered to be negligible, whereas a value higher than 1 × 10^−4^ was considered high risk and 10^−6^ < CR < 10^−4^ an acceptable/significant risk for human beings. The CR values from As, Cd, Cr and Pb for adults were 6.17 × 10^−6^, 1.16 × 10^−7^, 5.51 × 10^−6^ and 6.43 × 10^−8^, respectively. Likewise, the CR values of As, Cd, Cr and Pb for children were found to be 1.44 × 10^−5^, 2.70 × 10^−7^, 1.28 × 10^−5^ and 1.5 × 10^−7^, respectively. Overall, As and Cr showed significant carcinogenic risk for the populations associated with these two metals.

## 4. Conclusions

In this study, different pollution indices (EF, CF, Igeo, Ei, RI), principal component analysis and non-carcinogenic and carcinogenic methods were employed to investigate the pollution status, heavy metal sources and health risks associated with urban soil. Soil pH was near to neutrality, and EC and TDS were found to be normal and within the safe range (EC < 1000 µS/cm) in the water extract from the soil. Iron, Mn and Zn were the dominant metals in our study and, overall, the following decreasing trend was observed in park soils: Fe > Mn > Zn > Cr > Ni > Cu > Pb > As > Co > Cd > Tl > Hg. Compared with international agency guidelines (California Human Health (SSLs), Dutch Soil Guidelines, Canadian Soil Quality Guidelines, Spanish Government recommendations, the Government of Armenia), based on average values, all the studied HMs were lower than the guideline limits set by the agencies, except As and Cr. Arsenic mean levels and Cr levels were higher than the limits set by California Human Health (SSLs) in residential areas. When comparing the present results with worldwide levels, we found that Co, Cu, Hg, Tl, Zn, Ni, Pb and Fe levels were always lower than the reported worldwide values, while Cd and As levels were noticeably higher than some of the levels reported worldwide. The CVs of most metals showed low to moderate variation, except Pb and Hg, which showed CVs > 35%, indicating high variation, which might be associated with mixed sources. Principle component analysis was employed to assess the metal content relationships in the soil and the source apportionment of the metals. The Kaiser–Meyer–Olkin (KMO) test exhibited a good value (0.817), indicating that the HM data from the urban soils were suitable for PCA. The PCA indicated that Mn, Fe, Co, Ni, Cu, Zn, As, Cd, Pb, Cr and Tl may originate from natural sources, while the Hg, Pb, As and Cd showed anthropogenic influences and could be related to coal combustion, vehicle exhausts, automobile brakes, vehicle tires, pesticides and chemical fertilizers. The results for the Igeo showed that the soils were classified as not polluted to moderately polluted with Pb and Hg and moderately polluted with As and Cd. Based on the EF results, As and Cd exhibited significant enrichment on an average basis, while Pd and Hg also manifested significant enrichment in the maximum concentrations measured in the samples. Moreover, the contamination factor revealed that Zn and Pb showed moderate contamination, Hg exhibited low–moderate contamination and As and Cd showed high contamination in the soils. Similarly, the ecological risk assessment demonstrated that moderate risk was associated with As, while Cd was associated with high risk. The potential risk from heavy metals in the soils was measured using the potential ecological risk index, which revealed moderate risk from the metal levels in the soil on a cumulative basis. The non-carcinogenic risk and carcinogenic risk assessments for children and adults were determined using oral ingestion as the major exposure pathway for HMs in soils. Comparatively higher risk was noted for children. As was the major contributor (>50%) to non-carcinogenic risk, followed by Cr (>13%). The carcinogenic risk assessment of As, Cd, Cr and Pb indicated that As and Cr posed significant risks to the populations associated with urban soil.

## Figures and Tables

**Table 1:** Statistical distribution of physicochemical parameters and heavy metal levels (μg/g) in acid extracts of the soil.

		Mn	Fe	Co	Ni	Cu	Zn	As	Pb	Hg	Cr	Cd	Tl	pH	EC	TDS
Park Soils	Min	560.2	14,120	5.608	17.89	12.58	62.35	6.265	9.315	0.028	10.95	0.524	0.228	6.35	128.3	85.5
	Max	1129	24,209	8.801	32.34	21.84	104.5	12.20	51.63	0.179	33.34	0.809	0.440	7.03	1417	945.0
	Mean	818.3	21,011	6.849	23.63	17.23	79.99	8.965	17.20	0.062	24.28	0.665	0.365	6.75	336.1	223.1
	SD	137.2	2089	0.790	3.181	2.438	8.955	1.356	9.657	0.034	4.059	0.069	0.042	0.21	337.1	222.6
Residential Soils	Min	452.0	16,965	5.376	17.00	13.17	68.91	5.514	9.773	0.037	19.53	0.527	0.293	6.54	134.4	89.8
	Max	1114	24,056	8.064	31.40	22.53	89.79	11.99	31.36	0.111	27.56	0.809	0.473	7.05	1024	683.0
	Mean	783.5	20,896	6.708	24.80	17.38	81.58	9.147	14.67	0.060	23.62	0.670	0.377	6.70	281.9	187.9
	SD	191.3	2454	0.914	4.772	2.793	7.965	2.000	6.262	0.021	3.017	0.100	0.059	0.15	293.2	195.5

**Table 2:** Average metal concentrations (μg/g, dry weight) in soil in comparison with the worldwide reported levels and the international guideline values.

		As	Cd	Co	Cr	Cu	Fe	Hg	Mn	Ni	Pb	Tl	Zn	Acid Mixture	
Park soil		8.97	0.67	6.85	24.28	17.23	21,011	0.06	818.3	23.63	17.20	0.37	79.99	HNO_3_ + HCl	PS *
Residential soil		9.15	0.67	6.71	23.62	17.38	20,896	0.06	783.5	24.80	14.67	0.38	81.58	HNO_3_ + HCl	PS *
Jiaozuo city, China		55.81	-	20.21	51.80	20.36	-	-	460.77	34.43	27.56	-	84.21	HNO_3_ + HCl + HF + HClO_4_	[80]
Karachi, Pakistan		-	-	-	9.6	33.3	908.4	-	-	-	42.1	-	99.5	HNO_3_ + HClO_4_	[83]
Caserta, Italy		15.8	0.7	9.4	14.7	36	27,900	0.084	980	12.8	68	1.55	116.4	HNO_3_ + HCl	[84]
Isfahan city, Iran		16.17	2.17	13.15	80.57	92.75	-	-	-	61.65	179.97	-	470.36	HNO_3_ + HCl	[85]
Ghaziabad, India		-	0.4	-	288	122	21,433	-	386	147	147	-	187	HNO_3_ + HCl + H_2_O_2_	[86]
Yerevan, Armenia		0.776	-	13.7	122	103	36,400	0.115	776	61.9	110	-	262	X-ray fluorescence	[87]
Shanghai, China		8.3	0.3	-	115.3	40.4	-	0.2	-	37.4	45.0	-	144.8	HNO_3_ + HF + HClO_4_	[88]
Thiva, Greece		-	0.67	99	285	26	44,600	-	946	1777	30	-	78	HNO_3_ + HCl	[89]
Hualpén, Chile		3	-	15	15	24	-	-	-	33	6	-	918	HNO_3_ + HCl	[90]
Lisbon, Portugal		5.3	-	13.0	38	37	21,000	0.36	337	43	89	-	97	HNO_3_ + HCl	[91]
Niger, Nigeria		-	1.779	-	12.39	41.88	2038	-	211.1	42.54	915.8	-	63.37	HNO_3_	[92]
Chicago, USA		19.55	-	11	71	150	33,000	0.64	583	36	397	-	397	HNO_3_ + HCl + HF + HClO_4_	[93]
Baltimore, USA		-	1.06	15	72	45	23,495	-	472	27	231	-	141	HNO_3_ + HCl + H_2_O_2_	[94]
Oslo, Norway		5.48	0.41	9.98	32.5	31.7	21,582	0.13	486	28.4	55.6	-	160	HNO_3_	[95]
Darkhan, Mongolia		5.3	0.19	9.05	35	19.5	-	-	-	27.5	21.5	-	68.5	HNO_3_ + HCl + HF	[96]
	**Comparison with International Guidelines**														
California Human Health (SSLs)	Residential	0.07	1.7	660	17	3000	-	18	-	1600	150	5	23,000	-	[97]
	Commercial/industrial	0.24	7.5	3200	37	38,000	-	180	-	16,000	3500	63	100,000	-	[97]
Dutch Soil Guidelines	Background concentration	29	0.8	9	100	36	-	0.3	-	35	85	1	140	-	[98]
	Intervention value	55	12	240	380	190	-	10	-	210	530	-	720	-	[98]
	Max. permissible concentration (MPC)	34	1.6	33	100	40	-	2.2	-	38	140	1.3	160	-	[99]
Canadian Soil Quality Guidelines	Residential/parkland	12	10	-	64	63	-	6.6	-	50	140	1	200	-	[100]
Spanish Government recommendations	Regulatory guidance values	24	30	-	230	800	-	7	390	1560	270	3	11,700	-	[101]
RA Government, 2005		10	2	-	90	132	-	2.1	1500	80	65	-	220	-	[87]

PS *= Present study.

**Table 3:** Principal component analysis of selected metal levels in the soil.

	PC 1	PC 2	PC 3
Eigenvalue	7.04	1.26	1.02
Total variance (%)	58.6	10.5	8.50
Cumulative eigenvalue	7.04	8.29	9.31
Cumulative variance (%)	58.6	69.1	77.6
Mn	0.81	0.01	−0.30
Fe	0.90	0.06	0.21
Co	0.94	−0.02	−0.14
Ni	0.86	0.11	−0.33
Cu	0.84	−0.08	−0.17
Zn	0.78	−0.10	0.46
As	0.51	0.49	−0.20
Pb	0.48	−0.66	0.29
Hg	0.04	0.63	0.60
Cr	0.85	−0.22	0.17
Cd	0.79	0.33	0.02
Tl	0.89	−0.03	0.06

**Table 4:** Description of health risk assessment for heavy metals in soil.

			Adults		Children	
	RfD/RDA (mg/kg/day)	Conc.	HQ_ing_	CR_ing_	HQ_ing_	CR_ing_
As	0.0003	9.01	4.11 × 10^−2^	6.17 × 10^−6^	3.84 × 10^−1^	1.44 × 10^−5^
Cd	0.001	0.67	9.13 × 10^−4^	1.16 × 10^−7^	8.52 × 10^−3^	2.70 × 10^−7^
Co	0.02	6.81	4.67 × 10^−4^		4.36 × 10^−3^	
Cr	0.003	24.1	1.10 × 10^−2^	5.51 × 10^−6^	1.03 × 10^−1^	1.28 × 10^−5^
Cu	0.04	17.3	5.91 × 10^−4^		5.52 × 10^−3^	
Hg	0.0003	0.06	2.80 × 10^−4^		2.62 × 10^−3^	
Mn	0.14	809.6	7.92 × 10^−3^		7.39 × 10^−2^	
Ni	0.02	23.9	1.64 × 10^−3^		1.53 × 10^−2^	
Pb	0.004	16.6	5.67 × 10^−3^	6.43 × 10^−8^	5.29 × 10^−2^	1.5 × 10^−7^
Tl	0.00008	0.37	6.30 × 10^−3^		5.88 × 10^−2^	
Zn	0.3	80.4	3.67 × 10^−4^		3.43 × 10^−3^	
		HI	7.63 × 10^−2^		7.12 × 10^−1^	

## Data Availability

Not applicable.
